# Effectiveness of the universal prevention program 'Healthy School and Drugs': Study protocol of a randomized clustered trial

**DOI:** 10.1186/1471-2458-10-541

**Published:** 2010-09-08

**Authors:** Monique Malmberg, Geertjan Overbeek, Marloes Kleinjan, Ad Vermulst, Karin Monshouwer, Jeroen Lammers, Wilma AM Vollebergh, Rutger CME Engels

**Affiliations:** 1 Behavioural Science Institute, Radboud University Nijmegen, The Netherlands; 2 Developmental Psychology, Utrecht University, The Netherlands; 3 Trimbos Institute (Netherlands Institute of Mental Health and Addiction), Utrecht, The Netherlands; 4 Department of Interdisciplinary Social Science, Utrecht University, The Netherlands

## Abstract

**Background:**

Substance use is highly prevalent among Dutch adolescents. The Healthy School and Drugs program is a nationally implemented school-based prevention program aimed at reducing early and excessive substance use among adolescents. Although the program's effectiveness was tested in a quasi-experimental design before, many program changes were made afterwards. The present study, therefore, aims to test the effects of this widely used, renewed universal prevention program.

**Methods/Design:**

A randomized clustered trial will be conducted among 3,784 adolescents of 23 secondary schools in The Netherlands. The trial has three conditions; two intervention conditions (i.e., e-learning and integral) and a control condition. The e-learning condition consists of three digital learning modules (i.e., about alcohol, tobacco, and marijuana) that are sequentially offered over the course of three school years (i.e., grade 1, grade 2, and grade 3). The integral condition consists of parental participation in a parental meeting on substance use, regulation of substance use, and monitoring and counseling of students' substance use at school, over and above the three digital modules. The control condition is characterized as business as usual. Participating schools were randomly assigned to either an intervention or control condition.

Participants filled out a digital questionnaire at baseline and will fill out the same questionnaire three more times at follow-up measurements (8, 20, and 32 months after baseline). Outcome variables included in the questionnaire are the percentage of binge drinking (more than five drinks per occasion), the average weekly number of drinks, and the percentage of adolescents who ever drunk a glass of alcohol and the percentage of adolescents who ever smoked a cigarette or a joint respectively for tobacco and marijuana.

**Discussion:**

This study protocol describes the design of a randomized clustered trial that evaluates the effectiveness of a school-based prevention program. We expect that significantly fewer adolescents will engage in early or excessive substance use behaviors in the intervention conditions compared to the control condition as a direct result of the intervention. We expect that the integral condition will yield most positive results, compared with the e-learning condition and control condition.

**Trial registration:**

The protocol for this study is registered with the Nederlands Trial Register NTR1516

## Background

Dutch adolescents are one of the leaders in terms of drinking frequency and binge drinking in Europe and they usually start drinking in early adolescence [1]. Also, their use of tobacco and marijuana increases rapidly during this period [2]. This is worrisome in that early initiation of substance use has many detrimental consequences, such as distortion of brain development (e.g., [3]) and elevated risk for later dependence and misuse (e.g., [4]). Investigators and policy makers emphasize the importance of a delay in age of onset for preventing the adverse health consequences of early initiation of substance use.

The implementation of effective prevention programs is a potential powerful tool to lower the prevalence of substance use in early adolescents and to delay the age of onset of substance use. In the past, many school-based prevention programs have been developed and implemented [5-12]. In general, three major types of school-based interventions can be distinguished, namely knowledge, cognitive-affective, social influence, and alternative programs [6]. The knowledge programs aim to enhance students' knowledge on biological and psychological aspects of substance use in order to accomplish a more negative attitude towards substance use, which will deter actual use. The cognitive-affective programs argue that psychological factors place students in vulnerable positions and therefore aim to improve students' self-confidence and self-awareness. Finally, the social influence programs aim to improve social and/or life skills in order to prevent peer pressure leading to substance use. In the literature there is consensus on the fact that social influence programs seem to be most effective, in that they more often show positive effects compared to knowledge and affective programs [5,9,13,14]. Hence, previous studies showed that interactive methods sort more effect compared to non-interactive methods (e.g., [14,15]) in prevention of early and excessive substance use.

One of the most well-known and widely used universal prevention programs for Dutch early adolescents is 'The Healthy School and Drugs (HSD)' program. The HSD program combines elements of all three types of school-based prevention and is based on the ASE model [16-18], which is often used in predicting and explaining health behavior. The HSD program is annually implemented and carried out at approximately 60% of all secondary schools in The Netherlands, and is one of the few school-based Dutch prevention programs of which the effectiveness was studied in a quasi-experimental design [19]. The HSD program was mainly found to be effective on cognitive aspects (i.e., knowledge and attitude) of alcohol and tobacco use and less so on behavioral outcomes. Permanent improvement of the program and tuning to recent developments is essential and many changes were made in both materials (e.g., e-learning modules) and content (e.g., marijuana module) since the last evaluation. Hence, a recalibration of the effectiveness of the HSD program and its new materials seems necessary. Even more so, because the HSD program was never tested through a randomized controlled trial (e.g., [20]).

The HSD program is a multi-component prevention program aimed at reducing early and/or excessive substance use among adolescents. The program consists of four pillars, which are: information lessons (i.e., e-learning modules), parental participation, regulation of substance use, and monitoring and counseling of students' substance use. Although scholars argue that multi-component approaches, like the HSD program, are more effective than single component approaches (e.g., [15]) many Dutch schools do not want to invest time and resources in all components. To sort out if solely relying on the education of adolescents will have a preventive effect or that a multi-component approach of the HSD program is necessary in order to obtain such a preventive effect, we included an additional intervention condition (i.e., e-learning) in our study design.

### Aim and hypotheses

The primary aim of the 'Healthy School and Drugs' study is to assess the effectiveness of this multi-component universal prevention program by conducting a randomized clustered trial including 23 Dutch secondary schools. Three follow-up assessments (i.e., after 8, 20 and 32 months) will be carried out to examine the effects of the intervention conditions. Two hypotheses will be tested. First, in line with prior findings, we expect that the program will lead to a lower likelihood of unhealthy substance use behaviors. We expect that adolescents in the intervention conditions, relative to controls, will be less likely to engage in early or excessive substance use behaviors at follow-up. More specifically, we expect that this effect will be more pronounced in the integral condition compared to the control condition than in the e-learning condition compared to the control condition.

Second, following the ASE model, we expect that cognitive aspects of behavior will mediate the effects of the program. Specifically, we expect that adolescents included in the intervention conditions (as compared to controls) will (a) have more knowledge about the -harmful aspects of- specific substances, (b) have more negative and less positive attitudes towards substance use, (c) perceive to have less approval for using substances from their social environment, (d) have more confidence in refraining from use when confronted with tempting substance use offers, and (e) have more adequate risk perceptions concerning the consequences of substance use.

## Methods/Design

### Study design

The HSD effectiveness study is a 3-year randomized clustered trial (RCT) with three arms - two interventions (i.e., e-learning and integral) and a control condition - testing the prevention program effects. Participants are 3,784 early adolescents of 23 secondary schools from seven different regions in The Netherlands: 1,330 are involved in the e-learning condition, 1,195 in the integral condition, and 1,259 in the control condition. After initial recruitment and enrollment in the trial, randomization took place at the school level, to avoid contamination between conditions (e.g., [21]). Directly after conducting the randomization procedure, a baseline assessment will be carried out.

**Figure 1:**
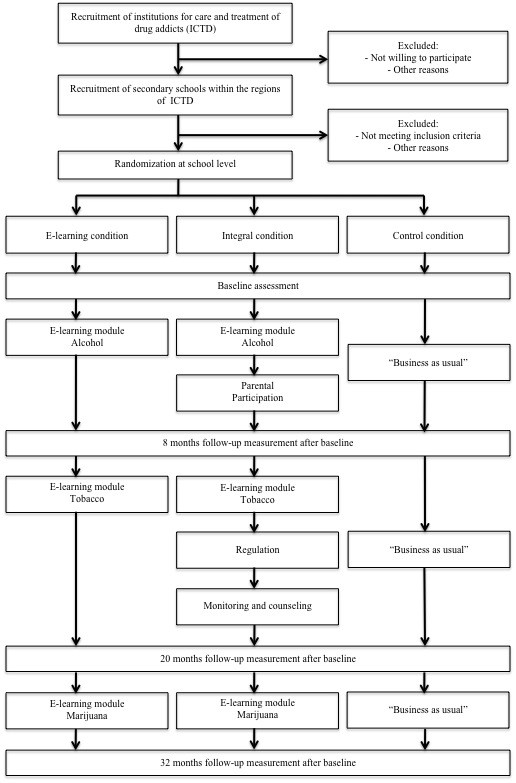
**Study design**.

The HSD program will be implemented after the baseline assessment in different phases to prevent overburdening of schools. In the first year - when adolescents are around 12 years old - the focus in the integral condition will be on starting with the information lessons on alcohol and to get parents involved in the program. In the second year, the schools in the integral condition will implement the two remaining pillars besides the information lessons on tobacco. Finally, in the third year the information lessons on marijuana will be implemented (see Figure 1). The schools in the e-learning condition will solely implement the information lessons on alcohol, tobacco, and marijuana in respectively the first, second, and third year. Finally, participants in the control condition will carry on in the same manner, thus a 'business as usual' approach will be followed at these schools.

Assessments in all three conditions will be conducted at baseline, after 8, 20, and 32 months. Participating schools receive the prevention program materials for free. Also, they receive school-based information about the substance use behaviors of their students after each assessment.

### Participants

#### Recruitment

The HSD program will be implemented in schools with the help of prevention departments of regional institutions for treatment and care of drug addiction (ITCD) and Municipal Health Services (MHS). Therefore, we contacted all these institutions to ask for corporation in the effectiveness trial. Seven institutions (i.e., VNN Friesland, VNN Groningen, VNN Drenthe, Centrum Maliebaan, GGD Zuid-Hollandse eilanden, Novadic-Kentron, and Mondriaan Zorggroep) agreed to take part in the study and secondary schools were recruited from these regions. All secondary schools in these regions were screened for eligibility. Exclusion criteria were recent involvement (i.e., parental participation in our target group, e-learning modules, regulation or monitoring and counseling activities in the past two years) in the HSD program and not offering a four-year education program. All eligible schools received an invitation letter and an information brochure and were contacted after two weeks to discuss participation in the study. Participants were thus recruited by school participation and all first grade students of participating secondary schools were included in the study. We visited the participating schools and during these visits further information was provided about the research project. In collaboration with the schools' headmasters, we informed the students' parents annually about the goals of the study by a letter in which parents were also notified that they could refuse participation of their child in the study. Thus, a passive informed consent procedure is followed in which parents (and their children) can refuse study participation by email, telephone or in person during the entire study period. Approval for the design and data collection procedures was obtained beforehand from the ethic committee of the Radboud University Nijmegen.

#### Randomization

Randomization occurred at the school level to avoid contamination between conditions. Thus, all first grade students from one school were allocated to the same condition (i.e., one of the intervention conditions or the control condition). An independent statistician performed the allocation before baseline assessment. Randomization was carried out centrally, using a blocked randomization scheme (block size 6) and stratified by level of education the schools offered.

### Sample size calculation

We estimated our targeted sample size based on a small effect size (*d*) of. 15 [22]. As the program has not been tested on effectiveness before, it is difficult to formulate an effect size. We based this on other prevention effectiveness studies on adolescent substance use, which generally have small effects [19]. We used the general-purpose statistical software package STATA to calculate the estimated sample sizes per condition. If a small effect occurs, then a sample size of N = 698 adolescents per condition is required at the end of the study for testing the hypothesis of superior effectiveness in a two-sided test at Alpha = 0.05 and a power of (1-Beta) = 0.80. We corrected this sample size for adolescents who will be lost in follow-up (e.g., changing schools, repeating grades) and for the fact that our data is clustered (i.e., adolescents are nested within schools). Considering these corrections, at least N = 1,061 adolescents per condition should be included to test the effectiveness of the HSD program. In accordance with the intention-to-treat principle, all adolescents randomized to a condition are included in further analyses testing the study hypotheses.

### Study intervention

#### Healthy School and Drugs program

The HSD program is a multi-component, school-based prevention program aimed at reducing excessive and early substance use among adolescents. The program for secondary schools consists of four pillars, which are:

1) Information lessons (e-learning modules): e-learning modules were developed for the information part of the program. These digital modules connect well to the experience of adolescents. Students work through the modules in their own pace during biology or counselor lessons, or in a special project week. The lessons pay attention to knowledge, attitude, and behavior with regard to substance use. Besides tutoring the students about the risks concerning substance use, students are also prepared for coping with group pressure by training their refusal skills. The modules consist of small films, animations, and several types of interactive tasks and identification is a central part of the modules. Also, adolescents are able to discuss relevant topics or to exchange their opinions through chat rooms and forums. Students receive three modules: alcohol (4 lessons), tobacco (3 lessons), and marijuana (3 lessons) in the first, second, and third grade, respectively. The lessons and modules are designed to gradually increase adolescent's skills in responsibly dealing with substances. Teachers are trained in the content and operation of the digital modules before the lessons are offered to the students.

2) Parental participation: Parents of first grade students are invited to attend a parental meeting in which information will be provided about the HSD prevention program and the relevant substances. Also, parents will be informed throughout a parental brochure and the school newsletter. The parental meeting will be held at school in collaboration with the ITCD or MHS. The duration of the parental meeting will be approximately 90 minutes. First, in a brief opening the attention of parents is captured by facts on substance use in adolescence. Then, brief information on the school regulation on substance use is provided. Characteristics and risks of substance use, opinions on substance use, and education in the home setting with respect to substance use will be discussed in the remainder of the meeting.

3) Regulation: the idea behind this pillar of the program is that rules set boundaries and create clarity. Therefore, the school needs to set an adequate regulation standard and rules concerning substance use behaviors of students and personnel. If the school lacks such regulation a special team will be instigated, including all relevant parties (e.g., parents, students, teachers, direction). This team will create or revise the rules and will plan how to communicate and maintain the rules in and around school. The school team will be assisted by the ITCD or MHS during this process.

4) Monitoring and counseling: An operation protocol (if absent) is to be formulated on how to deal with problematic substance use behaviors among students. Also, the ITCD or MHS will provide a training session on signaling and guiding problematic substance use among individual students. This training is meant for teachers, mentors, student speculators, and the care coordinator(s) of the school. During this training session practical information will be provided on how to recognize problematic use in students and on how to efficiently support these students. Further, advice will be given on how to use the operation protocol in daily practice.

#### Theoretical basis

The information lessons of the HSD program are based on the ASE model [16-18], which is commonly used in predicting and explaining health behavior. The ASE model is derived from the Theory of Reasoned Action (TRA; [23,24]) and the Social Cognitive Theory (SCT; [25]) and is based on the principles of student oriented tutoring [15]. Determinants of behavior, according to the ASE model, are attitudes (A), social influences (S), self-efficacy (E), and behavioral intention. Attitudes towards substance use behaviors result from outcome expectations of those specific behaviors. Other people's behaviors that directly or indirectly influence one's thoughts, feelings, and/or actions can be seen as social influence. Self-efficacy can be defined as one's experienced difficulty in refraining from using substances in tempting situations. Finally, intention is often assessed as the motivation or readiness to start using a specific substance in the future (e.g., [26]). The ASE model presumes that attitudes, social influence, and self-efficacy precede behavioral intentions. Also, the model assumes that behavioral intentions precede behavior.

The ASE components are imbedded in the e-learning modules and students work through these ASE components via the principles of information theory. First, effects of information lessons are only expected if students are tuned to the message, so the information should capture the attention of the students and should match the information needs of the students. Special triggers to capture students' attention (stories that students can identify with) are incorporated in the materials. Second, a message can only be effectively communicated if students understand the message. Information is therefore provided in a way that corresponds with the realm of adolescent thought by using age appropriate language and tasks. Third, becoming aware of attitudes and to influence these attitudes in the direction of the desired behavior, information is conceptualized as trustworthy and attuned to the student's opinions. Students are asked to think about pros and cons of substance use and are encouraged to make judgements themselves. Fourth, to account for the influence of the social environment, students are trained to resist social pressure. With the help of specific tasks on social norms, students are invited to think about social norms on substance use. Also, example videos are used to display how adolescents are influenced by others. Fifth, self-efficacy will influence actual behavior of students, thus students should be confident in their refusal skills. Students therefore learn how to carry out the desired behavior, and how to maintain and incorporate these behaviors into their daily living environment with the help of special processing tasks. Finally, students should persist in the desired behavior. Feedback on own behavior is important to achieve this goal, because it makes students aware of positive effects of their (changed) behavior. Students are challenged to think about what they will gain if they do not use a specific substance (just yet). All these information principles are processed in the design of the e-learning intervention by the following route: what happened (1), what do you know (2), what would you do (3 to 6). In total, adolescents work through this process three times; first for alcohol, then for tobacco, and finally for marijuana. Although the focus of the substance changes over the years, booster effects for the e-learning modules are expected because the training process is repeated.

The HSD prevention program is based on the assumption that more than information lessons are necessary to prevent adolescents from unhealthy substance use behaviors. Adolescents need rules and tutoring, a task for parents, school boards, teachers, and student counselors [9,14]. The HSD program therefore asks activities of all these parties (i.e., integral condition) and seeks synchronization of lessons, rules, and guidance.

#### Intervention conditions

The participating secondary schools were randomly assigned to one of the three following study conditions:

1) E-learning: Secondary schools that only carry out the e-learning modules. These schools will provide the information lessons in our target group.

2) Integral: Secondary schools that carry out the entire HSD program. These schools will also provide the information lessons in our target group, but also carry out the other three pillars of the HSD program.

3) Control: Secondary schools that do not carry out prevention activities. These schools are characterized by 'business as usual'. Many schools in The Netherlands have employed initiatives concerning substance use. The schools can carry on with these initiatives, as long as no HSD activities are carried out in our target group (both students and school personnel).

For the duration of our study, all participating schools, including those in the control condition, agreed not to implement or carry out other substance use prevention programs in the target group.

### Data collection

An overview of all measurements is given in Table 1. The baseline assessment took place in January-March 2009. During this measurement all first grade pupils of the school year 2008-2009 filled out a digital questionnaire during school hours in the presence of a teacher and a research assistant. The same procedure will be repeated three more times after the baseline assessment.

**Table 1:** Overview of measurements

Measurement	Baseline	Follow-up I (8 months after baseline)	Follow-up II (20 months after baseline)	Follow-up III (32 months after baseline)
Demographic characteristics	*	*	*	*
Alcohol:	*	*	*	*
Drinking behavior	*	*	*	*
Perceived parental rules	*	*	*	*
Intention	*	*	*	*
Global attitudes	*	*	*	*
Pros and cons	*	*	*	*
Social norm (approval)	*	*	*	*
Modeling	*	*	*	*
Self-efficacy	*	*	*	*
Knowledge	*	*	*	*
				
Tobacco:	*	*	*	*
Smoking behavior	*	*	*	*
Nicotine dependence	*	*	*	*
Perceived parental rules	*	*	*	*
Intention	*	*	*	*
Global attitudes	*	*	*	*
Pros and cons	*	*	*	*
Social norm (approval)	*	*	*	*
Modeling	*	*	*	*
Self-efficacy	*	*	*	*
Knowledge	*	*	*	*
				
Marijuana:	*	*	*	*
Marijuana using behavior	*	*	*	*
Marijuana dependence	*	*	*	*
Perceived parental rules	*	*	*	*
Intention	*	*	*	*
Global attitudes	*	*	*	*
Pros and cons	*	*	*	*
Social norm (approval)	*	*	*	*
Modeling	*	*	*	*
Self-efficacy	*	*	*	*
Knowledge	*	*	*	*
				
Other:	*	*	*	*
Risk perception	*	*	*	*
Popularity	*	*	*	*
Likability	*	*	*	*
Best friends	*	*	*	*
Personality	*	*	*	*
Pubertal development	*	*	*	*

#### Outcomes

The HSD prevention program targets reduction of substance use among adolescents. Since the program focuses on alcohol, tobacco, and marijuana use, we operationalized attainment targets for all three substances. The primary outcomes for alcohol are defined as the percentage of binge drinking (i.e., more than five drinks per occasion; [2]), the average weekly number of drinks [27], and the percentage of adolescents who ever drunk a glass of alcohol. The primary outcome with regard to tobacco and marijuana use is operationalized as the percentage of adolescents who ever smoked a cigarette or a joint, respectively [28,29]. The secondary outcome measure that we formulated for alcohol is the percentage of adolescents who drink on a weekly basis [27]. The percentage of adolescents who intend to smoke a cigarette or a joint in the future is defined as secondary outcome measure for tobacco and marijuana use, respectively [28,30]. Finally, to adequately test if potential effects of the HSD program are mediated by substance specific cognitions we also tapped adolescents' attitudes towards alcohol, tobacco, and marijuana as outcome measures (e.g., [31,32]). The same holds for social environment, self-efficacy, behavioral intentions, knowledge, and risk perception with respect to all three substances [1,17,33,34]. Other variables of interest, but no outcome measures, are perceived parental rules (on alcohol, tobacco, and marijuana; [35,36]), nicotine and marijuana dependence [1,37], popularity, likeability, friends, personality [38], and pubertal timing [39].

### Statistical analyses

Descriptive analyses will be conducted to check whether randomization has resulted in a balanced distribution of important student characteristics in all three conditions. Possible confounders will then be included in subsequent analyses to control for potential bias. Because the data have a multilevel structure (i.e., individuals are 'clustered' within schools), the possibility exists that the individual respondents are not independent within schools. To correct for the potential non-independence (complexity) of the data, the TYPE = COMPLEX procedure in Mplus will be used. This procedure corrects the standard errors of the parameter estimates for dependency leading to unbiased estimates.

For the main analyses, data will be analyzed in accordance with the intention-to-treat principle and in a completers-only framework by using Mplus [40], while controlling for sex, educational level, age, and ethnicity. Intention-to-treat means that all participants will be analyzed in the condition they were assigned to by randomization. Missing data will be handled by multiple imputation (MI), using the Markov Chain Monte Carlo (MCMC) method. Categorical variables are imputed with the help of logistic regression and ordinary regression is used for the imputation of the other variables. A total of 50 datasets will be completed by multiple imputation and prepared for data analyses in Mplus. Mplus will read the 50 datasets in via the TYPE = IMPUTATION option and will carry out the desired analyses for each dataset. Mediating the parameter estimates will then aggregate results for the 50 analyses. The standard errors of the parameter estimates are handled according to [41]. With respect to the completers-only analyses, only the participants with scores on all time points will be included. In both the intention-to-treat and the completers-only analyses the effects of the intervention conditions will be compared to the control condition. Both intervention conditions will individually be contrasted with the control condition.

### Time Frame

The recruitment, inclusion, and randomization of participants (i.e., schools) started in the fall of 2008. The final follow-up measurement is planned for the fall of 2011. The baseline data is collected between January and March 2009. The data of the follow-up measurements will be collected at three fixed time points. These assessments will take place between September and November 2009, September and November 2010, September and November 2011 respectively. Short-term results will be reported before the completion of the 32 months follow-up.

## Discussion

The present study protocol presents the design of a randomized clustered trial evaluating the effectiveness of the 'Healthy School and Drugs' prevention program. This universal prevention program aims at reducing excessive and early substance use in adolescence. It is hypothesized that adolescents in the intervention conditions will be less likely to engage in early or excessive substance use behaviors at follow-up compared to the control condition.

### Strengths and limitations

An important first strength of the Healthy School and Drugs program itself is that it is previously suggested that the program is partly effective in The Netherlands [19]. Another important strength is the clear and elaborate theoretical basis underlying the program. The ASE model [16-18] and information theory have been used to develop the e-learning modules. Also, the program consists of multiple components, which is in line with findings that multi-component programs sort more effects than single component programs (e.g., [15,21]). Finally, the program is a school-based prevention program, indicating that many adolescents will be reached when implemented.

A strength of the study design is that it does not only assess immediate effects, but also includes follow-up measurements at 8, 20, and 32 months. This allows us to test both the short and mid-term effects of the HSD program. Second, the extra study condition (i.e., the e-learning condition) will give the opportunity to optimally inform regional ITDC, MHS, and schools about the cost and benefits on their current prevention activities. Also, if the HSD program is found to be effective, the program might be (compulsory) implemented in more schools across The Netherlands, since present govern policy strongly encourages the implementation of effective intervention programs.

A limitation of the study is that information on the behavior of adolescents and their environment is entirely based on self-reports of the adolescents, which might lead to measurement errors. Two perspectives can explain possible measurement errors in self-reports on substance use, namely a situational and a cognitive perspective [42]. The situational perspective concerns the influence of the social environment, which might lead adolescents to give socially desirable answers. To avoid social desirability and optimize measurement validity, we will guarantee full confidentiality (anonymity) to our participants (e.g., [43]). The cognitive perspective concerns the cognitive or internal processes that might influence the self-reports. Adolescents might over or underestimate their substance use behaviors in that they can not exactly recall what they have been using in a certain period (e.g., [44]). In our study we will ask participants if they ever tried a specific substance, which is arguably different from asking them how much they have used in a certain period. One might expect participants to reliably recall ever using alcohol, tobacco, or cannabis before. With respect to the questions on use during a certain period, the cognitive aspect seems more relevant, thus one might argue that more measurement errors will occur in these self-reports. However, the time between the period and assessment seems to matter. The longer the time interval the more severe recall bias one might expect (e.g., [44,45]). In our study, the time interval is relatively short (past month or past week), which will optimize the reliability of the self-reports.

### Implications for practice

Based on the results of the HSD effectiveness study, the prevention program will be adjusted accordingly. If necessary the content of the program will be renewed, as will the theoretical concepts and the different parts (i.e., pillars) of the prevention program. In short, the results will drive the (re)development of the HSD program in the next couple of years.

## Conclusion

This study will evaluate a multi-component school-based prevention program on substance use in adolescence. The results of this study will provide insights into the effectiveness of the Healthy School and Drugs prevention program and the precursors of substance use among Dutch early adolescents.

## Competing interests

Karin Monshouwer and Jeroen Lammers work for the Trimbos-institute, which is the institution that developed the 'Healthy School and Drugs' project. Also, this institution is principal for the effectiveness study. The other author(s) declare that they have no competing interests.

## Authors' contributions

MM is responsible for the data collection, data analysis and for reporting the study results. All others authors are supervisors and grant applicators. All authors read and approved the final manuscript.

## Pre-publication history

The pre-publication history for this paper can be accessed here:

http://www.biomedcentral.com/1471-2458/10/541/prepub
